# Supplementary material for: Empirical methods for controlling false positives and estimating confidence in ChIP-Seq peaks
Source: BMC Bioinformatics. 2008 Dec 5;9:523. doi: 10.1186/1471-2105-9-523 (PMC2628906; doi:10.1186/1471-2105-9-523)
Supplement: Additional File 1 — A variety of html documents from the USeq web site detailing the available applications, their best usage, output file type descriptions, command line menus, etc. [file 1471-2105-9-523-S1.zip › USeqUserGuides/JavaDoc.zip!/JavaDoc/bioroot/class-use/IdNameMatch.html]

Uses of Class bioroot.IdNameMatch


|  |  |  |
| --- | --- | --- |
| **Overview** \| **Package** \| **Class** \| **Use** \| **Tree** \| **Deprecated** \| **Index** \| **Help** | |  |
| PREV   NEXT | **FRAMES**    **NO FRAMES**     **All Classes** |  |


---


## **Uses of Class bioroot.IdNameMatch**

| Packages that use IdNameMatch | |
| --- | --- |
| **bioroot** |  |
| **bioroot.oligo** |  |
| **bioroot.plasmid** |  |
| **bioroot.strain** |  |

Uses of IdNameMatch in bioroot

| Methods in bioroot that return IdNameMatch | |
| --- | --- |
| `IdNameMatch[]` | `DBUtil.fetchIdNameMatchArray(java.lang.String sql)`             Fetches an IndexNameMatch Array. |
| `static IdNameMatch` | `IdNameMatch.findMatch(IdNameMatch[] inm, java.lang.String test)` |

| Methods in bioroot with parameters of type IdNameMatch | |
| --- | --- |
| `static IdNameMatch` | `IdNameMatch.findMatch(IdNameMatch[] inm, java.lang.String test)` |

Uses of IdNameMatch in bioroot.oligo

Constructors in bioroot.oligo with parameters of type IdNameMatch

`OligoBean(java.lang.String[] cells,
IdNameMatch[] genes,
IdNameMatch[] organisms,
UserBean userBean,
DBUtil bioRoot)`
  
          For bulk file upload should be 17? cells
Name, Alias Oligo Sequence, Matching Gene Sequence, uM Stock Concentration, ng/uL Stock Concentration, Volume(uL), Purification,
0 1 2 3 4 5 6 7
Modifications, Orientation, Type, Storage Location, Availibility, VisibilityBean, Handling Instructions, Target Gene Name, Organism, Notes
8 9 10 11 12 13 14 15 16 17

`OligoBean(java.lang.String[] cells,
IdNameMatch[] genes,
IdNameMatch[] organisms,
UserBean userBean,
DBUtil bioRoot)`
  
          For bulk file upload should be 17? cells
Name, Alias Oligo Sequence, Matching Gene Sequence, uM Stock Concentration, ng/uL Stock Concentration, Volume(uL), Purification,
0 1 2 3 4 5 6 7
Modifications, Orientation, Type, Storage Location, Availibility, VisibilityBean, Handling Instructions, Target Gene Name, Organism, Notes
8 9 10 11 12 13 14 15 16 17

Uses of IdNameMatch in bioroot.plasmid

Constructors in bioroot.plasmid with parameters of type IdNameMatch

`PlasmidBean(java.lang.String[] cells,
IdNameMatch[] genes,
IdNameMatch[] organisms,
IdNameMatch[] markerIDMatch,
UserBean userBean,
DBUtil bioRoot)`
  
          For bulk file upload should be 16 cells
0 1 2 3 4 5 6 7 8 9 10 11 12 13 14 15 16
Name Alias Source Recipients Reference Gene Organism Markers StrainIds Location Concentration Volume Purification Availability VisibilityBean Notes Sequence

`PlasmidBean(java.lang.String[] cells,
IdNameMatch[] genes,
IdNameMatch[] organisms,
IdNameMatch[] markerIDMatch,
UserBean userBean,
DBUtil bioRoot)`
  
          For bulk file upload should be 16 cells
0 1 2 3 4 5 6 7 8 9 10 11 12 13 14 15 16
Name Alias Source Recipients Reference Gene Organism Markers StrainIds Location Concentration Volume Purification Availability VisibilityBean Notes Sequence

`PlasmidBean(java.lang.String[] cells,
IdNameMatch[] genes,
IdNameMatch[] organisms,
IdNameMatch[] markerIDMatch,
UserBean userBean,
DBUtil bioRoot)`
  
          For bulk file upload should be 16 cells
0 1 2 3 4 5 6 7 8 9 10 11 12 13 14 15 16
Name Alias Source Recipients Reference Gene Organism Markers StrainIds Location Concentration Volume Purification Availability VisibilityBean Notes Sequence

Uses of IdNameMatch in bioroot.strain

Constructors in bioroot.strain with parameters of type IdNameMatch

`StrainBean(java.lang.String[] cells,
IdNameMatch[] cellTypes,
IdNameMatch[] organisms,
IdNameMatch[] markerIDMatch,
UserBean userBean,
DBUtil bioRoot)`
  
          For bulk file upload should be 23 cells
0 Name
1 Alias
2 Source
4 Reference
5 Type Bacteria, Yeast, Worm, Insect, Plant, Fish, Cell Line, Bird, Mammal or Other
6 Recipients
7 Organism name
8 Markers names, comma seperated
9 Plasmid Ids
10 Parent Strain Ids
11 Storage Location
12 Mutagen EMS, ENU, MMS, MNU, X-ray, UV, Transposon, T-DNA, Spontaneous or Other
13 Genotype/ Background
14 Phenotype
15 Growth Conditions
16 Restock
17 Remaining Aliquots
18 Array Type Extra Chromosomal or Integration
19 Seed Type Individual Line, Individual Pool, Set of Lines, or Set of Pools
20 Cell Type (Cell Lines)
21 Passage Number ( Cell Lines)
22 Mating Type ( Yeast) diploid, a, alpha, h+, or h-)
23 Availability
24 VisibilityBean
25 Notes

`StrainBean(java.lang.String[] cells,
IdNameMatch[] cellTypes,
IdNameMatch[] organisms,
IdNameMatch[] markerIDMatch,
UserBean userBean,
DBUtil bioRoot)`
  
          For bulk file upload should be 23 cells
0 Name
1 Alias
2 Source
4 Reference
5 Type Bacteria, Yeast, Worm, Insect, Plant, Fish, Cell Line, Bird, Mammal or Other
6 Recipients
7 Organism name
8 Markers names, comma seperated
9 Plasmid Ids
10 Parent Strain Ids
11 Storage Location
12 Mutagen EMS, ENU, MMS, MNU, X-ray, UV, Transposon, T-DNA, Spontaneous or Other
13 Genotype/ Background
14 Phenotype
15 Growth Conditions
16 Restock
17 Remaining Aliquots
18 Array Type Extra Chromosomal or Integration
19 Seed Type Individual Line, Individual Pool, Set of Lines, or Set of Pools
20 Cell Type (Cell Lines)
21 Passage Number ( Cell Lines)
22 Mating Type ( Yeast) diploid, a, alpha, h+, or h-)
23 Availability
24 VisibilityBean
25 Notes

`StrainBean(java.lang.String[] cells,
IdNameMatch[] cellTypes,
IdNameMatch[] organisms,
IdNameMatch[] markerIDMatch,
UserBean userBean,
DBUtil bioRoot)`
  
          For bulk file upload should be 23 cells
0 Name
1 Alias
2 Source
4 Reference
5 Type Bacteria, Yeast, Worm, Insect, Plant, Fish, Cell Line, Bird, Mammal or Other
6 Recipients
7 Organism name
8 Markers names, comma seperated
9 Plasmid Ids
10 Parent Strain Ids
11 Storage Location
12 Mutagen EMS, ENU, MMS, MNU, X-ray, UV, Transposon, T-DNA, Spontaneous or Other
13 Genotype/ Background
14 Phenotype
15 Growth Conditions
16 Restock
17 Remaining Aliquots
18 Array Type Extra Chromosomal or Integration
19 Seed Type Individual Line, Individual Pool, Set of Lines, or Set of Pools
20 Cell Type (Cell Lines)
21 Passage Number ( Cell Lines)
22 Mating Type ( Yeast) diploid, a, alpha, h+, or h-)
23 Availability
24 VisibilityBean
25 Notes

---


|  |  |  |
| --- | --- | --- |
| **Overview** \| **Package** \| **Class** \| **Use** \| **Tree** \| **Deprecated** \| **Index** \| **Help** | |  |
| PREV   NEXT | **FRAMES**    **NO FRAMES**     **All Classes** |  |


---
